# Expression of surfactant protein D in airways of asthmatics and interleukin-13 modulation of surfactant protein D in human models of airway epithelium

**DOI:** 10.1186/s12931-015-0177-7

**Published:** 2015-02-15

**Authors:** Jie Xu, Gurpreet K Singhera, Delbert R Dorscheid

**Affiliations:** Center for Heart Lung Innovation, University of British Columbia and St. Paul’s Hospital, Vancouver, British Columbia

**Keywords:** Surfactant protein D, Asthma, Airway epithelium, Air-liquid-interface, Interleukin-13

## Abstract

**Background:**

Surfactant protein D (SP-D), a pattern recognition molecule, has been shown to play roles in host defense such as opsonisation, aggregation of pathogens, and modulation of the inflammatory response. In light of infection-induced exacerbations and damage to the airway epithelium from inflammation, these functions of SP-D make it relevant in the development and pathogenesis of asthma.

**Methods:**

Expression of SP-D was examined in human airway sections and primary airway epithelial cells (AEC) grown in air-liquid interface (ALI) cultures and comparisons were made between those from asthmatic and non-asthmatic donors. ALI cultures of AEC from non-asthmatic donors were examined for SP-D, Mucin 5AC, and cytokeratin-5 expression at different stages of differentiation. Interleukin-13 (IL-13) treatment of airway epithelium and its effect on SP-D expression was studied using ALI and monolayer cultures of primary AEC from non-asthmatic and asthmatic donors.

**Results:**

Airway epithelium of asthmatics, compared to that of non-asthmatics, expressed increased levels of SP-D as demonstrated in airway tissue sections (fraction of epithelium 0.66 ± 0.026 vs. 0.50 ± 0.043, p = 0.004) and ALI cultures (fraction of epithelium 0.50 ± 0.08 vs. 0.25 ± 0.07). SP-D expression decreased as ALI cultures differentiated from 7 days to 21 days (fraction of epithelium 0.62 ± 0.04 to 0.23 ± 0.03, p = 0.004). Treatment with IL-13 decreased SP-D expression in both ALI cultures (fraction of epithelium 0.21 ± 0.06 vs. 0.62 ± 0.04, p = 0.0005) and monolayer cultures (protein expression fold change 0.62 ± 0.05) of non-asthmatic AEC; however, IL-13 had no significant effect on SP-D expression in monolayer cultures of asthmatic AEC. Experiments with non-asthmatic monolayer cultures indicate IL-13 exert its effect on SP-D through the IL-13 receptor alpha1 and transcription factor STAT6.

**Conclusions:**

SP-D is expressed differently in airways of asthmatics relative to that of non-asthmatics. This can have implications on the increased susceptibility to infections and altered inflammatory response in asthmatic patients. Future functional studies on the role of SP-D in asthma can provide better insight into defects in the structure and regulation of SP-D.

**Electronic supplementary material:**

The online version of this article (doi:10.1186/s12931-015-0177-7) contains supplementary material, which is available to authorized users.

## Introduction

Asthma is a chronic inflammatory disorder of the airways which affects people of all ages. It has increased in prevalence over the past 30 years and currently affects 235 million people worldwide [1]. Clinically, asthma is characterized by recurrent and reversible episodes of wheezing, chest tightness, breathlessness, and coughing. Structural and functional changes associated with asthma include bronchoconstriction, airway inflammation, epithelial goblet cell hyperplasia, bronchial smooth muscle hypertrophy, and proliferation of airway blood vessels and nerves [2]. Higher proportion of basal cells as opposed to differentiated cells has also been found in airways of asthmatics [3].

Asthmatic patients may have increased susceptibility to viral and bacterial infections in the airways. Respiratory viral infections have detrimental effects on patients with established asthma; they have been associated with almost 80% of asthma exacerbations [4]. Respiratory syncytial virus (RSV) is the most common cause of acute lower respiratory infection in infants and children worldwide [5]. Several studies have demonstrated an association of severe RSV infection in infancy with subsequent development of recurrent wheezing and asthma in children [6-9]. Asthma has traditionally been closely associated with the adaptive immune system; however, more recent studies have indicated that the innate immune response is also important in its development and progression [10]. Therefore, the role of the innate immune system in host defence against inhaled pathogens and subsequent inflammatory response is of particular interest.

Pulmonary surfactant was originally identified as a lipoprotein complex which reduces surface tension in the alveoli. More recently, the surfactant proteins A and D (SP-A, SP-D), were found to be involved in host defense mechanisms [11]. SP-A and SP-D, belong to the collectin family of proteins and are pattern recognition molecules which bind a broad spectrum of pathogens including viruses, bacteria, and fungi [11]. Its roles in host defense include enhancement of phagocytosis through opsonisation, aggregation of pathogens, and regulation of inflammatory mediators [11]. SP-A and SP-D are constitutive mediators of antigen clearance which can interact with cellular components of both innate and adaptive immune system on the mucosal surface [12].

Interleukin-13 (IL-13) is a cytokine secreted by many cell types including T_H_2 cells, macrophages, and activated mast cells [13-15]. Several animal models have indicated that IL-13 mediates features of asthma such as airway hyper-reactivity, mucus cell hyperplasia, and sub-epithelial airway fibrosis independent of other cytokines [13,16,17]. The role of IL-13 in mediating phenotypes of asthma was also supported by reports of increased IL-13 in lungs of asthmatic patients [18]. IL-13 has two receptors: IL-13 receptor alpha 1 (IL-13Rα1) and IL-13 receptor alpha 2 (IL-13Rα2). IL-13Rα1 forms a heterodimeric receptor complex with IL-4Rα subunit. IL-13 signalling via IL-13Rα1 activates the Janus kinase (JAK)-signal transducer and activator of transcription (STAT) pathway, specifically the transcription factor STAT6. IL-13Rα2 is known as a high-affinity receptor and can signal via the transcription factor, activator protein 1[19].

The present study aims to characterize the expression of SP-D in airways of asthmatics, with the hypothesis that it would differ from that of non-asthmatics. The effects of differentiation and IL-13 exposure on the expression of SP-D were investigated using air-liquid interface (ALI) cultures of primary airway epithelial cells (AEC) from non-asthmatic donors and monolayer cultures of AEC from non-asthmatic and asthmatic donors.

## Material and methods

### Reagents

Mouse anti-human SP-D antibody used for immunohistochemistry (IHC) and Western blot analysis (catalog no. HYB-245-01-02) was purchased from Thermo Scientific (Rockford, IL). Mouse anti-β-actin monoclonal antibody conjugated to horseradish peroxidase (sc-47778) was purchased from Santa Cruz Biotechnology (Santa Cruz, CA). Mouse anti-Mucin 5AC (MUC5AC) antibody (AB24071) and rabbit anti-cytokeratin 5 (CK-5, AB24647) antibody were purchased from Abcam Inc. (Toronto, ON) for IHC. Recombinant human IL-13 (213-IL), goat anti-human IL-13Rα1 (AF-152) and IL-13Rα2 (AF-146) neutralizing antibodies and normal goat IgG (AB-108-C) were purchased from R&D Systems (Minneapolis, MN). Mouse anti-phosphorylated STAT6 (p-STAT6) antibody (611566) and mouse anti-STAT6 antibody (611290) used for Western blotting were purchased from BD Biosciences (Mississauga, ON). Trizol reagent (15596018) was purchased from Life Technologies (Burlington, ON).

### Cell culture

Human lungs of de-identified asthmatic and non-asthmatic donors, deemed unsuitable for transplantation and donated to medical research, were obtained from the International Institute for the Advancement of Medicine (Edison, NJ) for primary cell isolation as per approval by the Research Ethics Board (REB) of University of British Columbia/Providence Healthcare (REB# H00-50110). Airway epithelial cells (AEC) were isolated by protease digestion as described by Gray and colleagues [20], cultured in bronchial epithelial growth medium (Lonza, Mississauga, ON, CC-3170), incubated at 37°C in 5% CO_2,_ and grown in 12-well plates. Treatment of primary AEC in monolayer occurred between passages two and three. Donor-matched air-liquid interface (ALI) epithelial cultures were generated using cells at passages one or two in PneumaCult medium (Stemcell Technologies, Vancouver, BC, 05001). ALI epithelial cultures were grown for 7, 14, or 21 days to represent various stages of differentiation.

### Stimulation of cell culture

Primary AEC from non-asthmatic donors were grown to 80% confluence and treated for 24 hours with IL-13 (10 ng/mL) alone or pre-incubated with either goat IgG isotype control, neutralizing antibodies for IL-13Rα1 (20 μg/mL) or IL-13Rα2 (2 μg/mL) for one hour prior to the addition of IL-13. Primary AEC from asthmatic donors were similarly grown to 80% confluence and treated for 24 hours with IL-13 (10 ng/mL).

Pseudostratified ALI cultures of AEC isolated from non-asthmatic donors were grown for 28 days to study the effects of IL-13 treatment. Starting at days 14 and 21, IL-13 was added on alternate days for the duration of two weeks and one week respectively.

### Immunohistochemical analysis

Human airway tissue from asthmatic and non-asthmatic donors and ALI cultures grown from non-asthmatic AEC (untreated and IL-13 treated) were fixed in 10% formalin, embedded in paraffin, and sectioned into 4 μm slices. Sections were deparaffinized in CitriSolv (Fisher Scientific, Toronto, ON, 22-143-975) and rehydrated. Antigen retrieval was performed by autoclaving in Citra solution at pH 6.0 (Life Technologies, Burlington, ON) for 22 minutes. All sections were blocked consecutively using Background Sniper (Biocare Medical, Markham, ON, B5966L) and Dual Endogenous Block (Dako, Burlington, ON, K5361). Sections were stained with anti-SP-D (0.25 μg/mL), MUC5AC (1 μg/mL), or CK-5 (1 μg/mL) antibodies using biotin-free MACH 3 AP-Polymer Detection kit (Biocare Medical, Markham, ON, M3U532 for mouse and M3R533 for rabbit primary antibodies) containing alkaline phosphatase with Warp Red Chromogen (Biocare Medical, Markham, ON, WR806) as substrate. Matched isotype control antibodies were used as negative controls for IHC to demonstrate antibody specificity. Hematoxylin was used for counter-staining of the nuclei. Colour segmentation via ImagePro Plus (Media Cybernetics, Silver Spring, MD) was performed for quantification whereby area positive was normalized to total epithelial area.

### Western blot analysis

Total cell lysates collected from primary human AEC monolayers and ALI cultures were used for Western blot analysis according to standard techniques [21]. Twenty micrograms of reduced protein samples were electrophoresed in a 4-20% gradient SDS-PAGE gel and then transferred to a nitrocellulose membrane. The membrane was blocked for 1 hour at room temperature in TBST (10 mmol/L Tris-HCl, 150 mmol/L NaCl, and 0.1% Tween-20) containing 5% skimmed milk for non-phosphorylated proteins and 5% BSA for phosphorylated proteins. The membrane was then incubated overnight at 4°C in respective primary antibodies in TBST containing 2.5% skimmed milk for non-phosphorylated protein or 1% BSA for phosphorylated protein. Primary antibody concentrations used were: anti-SP-D (1:1000) and anti-p-STAT6 (1:2000) followed by re-probing with anti-β-actin (1:2500) and STAT6 (1:1000) to normalize for SP-D and p-STAT6 respectively. The membranes were washed three times with TBST, followed by incubation with a 1:2000 dilution of horseradish peroxidase-labeled goat anti-mouse IgG (BD Biosciences Canada, Mississauga, ON). Samples were detected with enhanced chemiluminescence Super Signal West Femto (Pierce, Cheshire, United Kingdom). Densitometry was performed via ImageJ (National Institutes of Health, Bethesda, MD) for quantification. In experiments with multiple treatments, densitometry values for SP-D were normalized first to β-actin and then to untreated control to obtain fold change protein expression of SP-D.

### Quantitative real time PCR

Total RNA was isolated using Trizol reagent according to manufacturer’s protocol, and 0.6 μg of total RNA was converted to complementary DNA (cDNA) through reverse transcription (RT) using qScript cDNA SuperMix from Quanta Biosciences (Gaithersburg, MD). RT reaction was incubated at 25°C for 5 minutes, 42°C for 30 minutes, and 85°C for 5 minutes. Quantitative real-time PCR (qPCR) was performed on ABI ViiA 7 (Life Technologies, Burlington, ON) with 20 μL reactions containing 1 μL of cDNA, 5 μL of TaqMan master mix (Life Technologies, Burlington, ON), and 0.5 μl of SP-D TaqMan assay probe (Hs00358340_m1 SFTPD) or hypoxanthine phosphoribosyltransferase (HPRT) TaqMan assay probe (Hs99999909_m1HPRT1) in 384-well plates. qPCR conditions were one cycle of 50°C for 2 minutes, one enzyme activation cycle of 95°C for 10 minutes, and 40 cycles of amplification. Each amplification cycle consists of denaturation at 95°C for 15 seconds, followed by simultaneous annealing and extension at 60°C for 1 minute. Results were analyzed with ViiA 7 software using the comparative quantification method where CT values of SP-D were normalized to CT values of HPRT followed by normalization to untreated control samples to obtain ΔΔ_CT_ = [(_CT_SP-D – _CT_HPRT)_Treated_ – (_CT_SP-D – _CT_HPRT)_Untreated_]. Expression fold change was defined as 2^-ΔΔCT^.

### Statistical analysis

GraphPad Prism 5 (GraphPad Software, San Diego, CA) was used for all statistical analyses. Colour segmentation data from human airway sections and baseline ALI sections between asthmatic and non-asthmatic donors were analyzed using a two-tailed, unpaired *t* test while data from IL-13 treated ALI sections was analyzed using two-tailed, paired *t* test. Data from IL-13 treated asthmatic and non-asthmatic monolayer cultures was also analyzed using a two-tailed, paired *t-*test against the respective controls. Data from ALI cultures that differentiated from 7 to 21 days was analyzed by one-way ANOVA followed by Bonferroni’s Multiple Comparison Test and post-test for linear trend where appropriate. Densitometry data from IL-13 treated non-asthmatic monolayer cultures (with and without neutralizing antibodies) was analyzed by one-way ANOVA with Bonferroni’s Multiple Comparison Test. Values are presented as mean ± SEM. Statistical significance is defined as p-value < 0.05.

## Results

### SP-D expression in human airway sections

**Figure 1:**
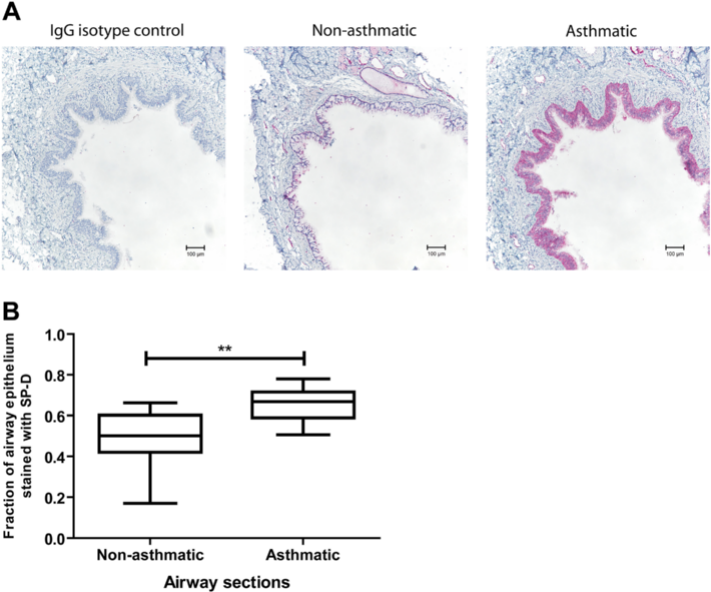
**Surfactant protein D expression in human airway sections.** SP-D expression was examined in sections of airways from non-asthmatic and asthmatic donors via immunohistochemistry, where pink was indicative of positivity **[A]**. Fraction of total epithelial area positive was quantified using colour segmentation in ImagePro Plus. Expression of SP-D in the airway epithelia of asthmatic donors was significantly higher compared to non-asthmatic donors (n = 11, t-test p = 0.004) **[B]**.

Lung tissue sections obtained from 11 asthmatic and 11 non-asthmatic donors were used to characterize the expression of SP-D in human airways. Immunohistochemistry was performed to study the distribution and relative quantity of SP-D expression. Airways of both asthmatic and non-asthmatic donors expressed SP-D in the cytoplasm of epithelial cells (Figure 1A). When positive staining was expressed as a fraction of total epithelial area, asthmatic airways demonstrated higher SP-D expression (0.66 ± 0.026) compared to non-asthmatic airways (0.50 ± 0.043) (n = 11, p = 0.004, Figure 1B). The presence of SP-D was confirmed in human airways and further, its expression was increased in airways of asthmatics. The non-uniform intensity indicates that SP-D may be expressed more in certain subtypes of airway epithelial cells, specifically undifferentiated basal cells.

### SP-D expression in human air-liquid interface cultures

**Figure 2:**
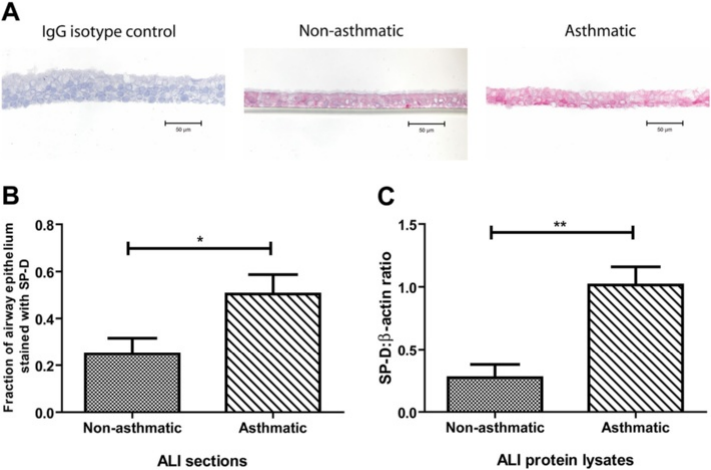
**Surfactant protein D expression ALI cultures.** SP-D expression was examined in ALI sections grown from non-asthmatic and asthmatic donors via immunohistochemistry, where pink was indicative of positivity **[A]**. Fraction of total epithelial area positive was quantified using colour segmentation in ImagePro Plus. Expression of SP-D in the ALIs of asthmatic AEC was significantly higher compared to that of non-asthmatic AEC (n = 5, t-test p = 0.04) **[B]**. SP-D protein expression in total cell lysates of ALI cultures was analyzed by Western blot. Expression of SP-D was quantified by densitometry using ImageJ and normalized to β-actin. ALIs of asthmatic AEC demonstrated significantly higher expression of SP-D compared to that of non-asthmatic AEC (n = 3-7, t-test p = 0.005) **[C]**.

Well-differentiated twenty-one day ALI cultures of primary asthmatic AEC demonstrated higher levels of SP-D expression compared to that of non-asthmatics as detected by immunohistochemistry (fraction of total epithelial area: 0.50 ± 0.08 vs. 0.25 ± 0.07, n = 5, p = 0.04, Figure 2A, B). Western blotting also demonstrated higher SP-D expression in ALI cultures derived from asthmatic AEC (1.02 ± 0.15, n = 3) compared to those from non-asthmatic AEC (0.27 ± 0.11, n = 7, p = 0.005, Figure 2C). These findings are in concordance with those of airway tissue sections and suggest that ALI epithelial cultures are a viable model for studying the expression of SP-D.

### SP-D expression and differentiation of ALI cultures

**Figure 3:**
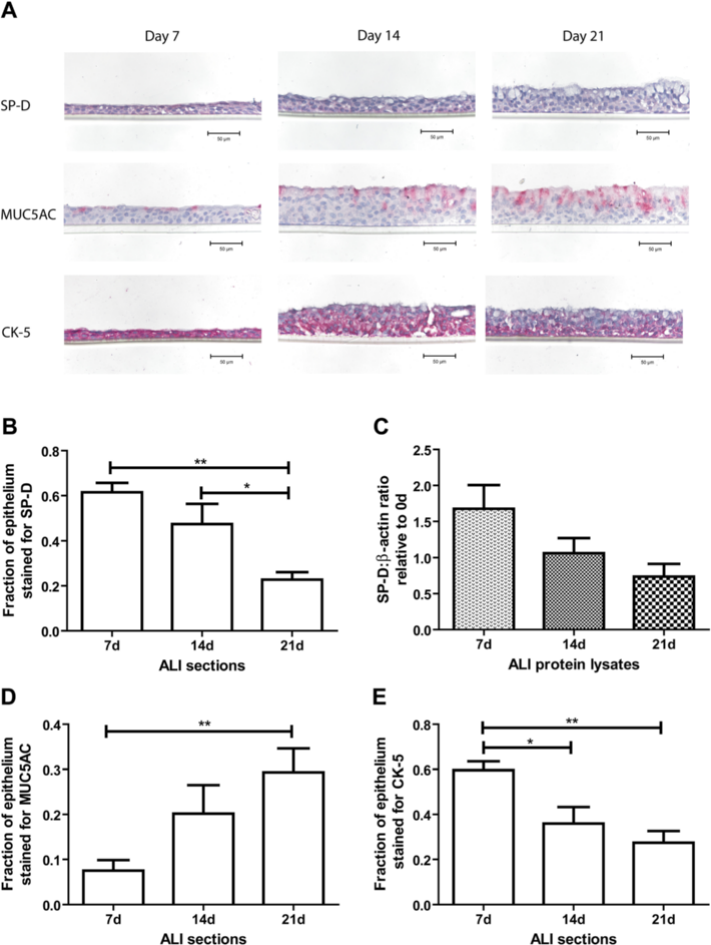
**Surfactant protein D, Mucin 5AC and cytokeratin-5 expression in ALI cultures at different differentiation stages.** SP-D, MUC5AC, and CK-5 expression were examined in ALI sections grown for 7, 14, and 21 days from non-asthmatic donors via immunohistochemistry, where pink was indicative of positivity **[A]**. Fraction of total epithelial area positive was quantified using colour segmentation in ImagePro Plus. Expression of SP-D in the airway epithelia decreased significantly as the cultures differentiated from day (d) 7 to 21 (n = 5, one-way ANOVA p = 0.004) **[B]**. SP-D protein expression in 7, 14, and 21-day ALI cultures were detected by Western blotting, quantified by densitometry relative to cultures at 0 days. A linear trend was observed (n = 3, p = 0.04) **[C]**. Expression of MUC5AC in the airway epithelia increased significantly as the cultures grew from day 7 to 21 as demonstrated by immunohistochemistry (n = 4, one-way ANOVA p = 0.007) **[D]**. Expression of CK-5 in the airway epithelia decreased significantly as the cultures grew from day 7 to 21 (n = 4, one-way ANOVA p = 0.002) **[E]**.

ALI cultures grown from non-asthmatic AEC were exposed to air and allowed to differentiate for 7, 14, and 21 days. The fraction of epithelium stained positive for SP-D significantly decreased from 0.62 ± 0.04 at 7 days to 0.47 ± 0.09 at 14 days and to 0.23 ± 0.03 at 21 days, as detected by immunohistochemistry (n = 5, p = 0.004, Figure 3A, B). Similarly, the fold change of SP-D protein expression relative to day 0 decreased from 1.68 ± 0.33 at 7 days to 1.06 ± 0.21 at 14 days and to 0.74 ± 0.18 at 21 days as detected by Western blotting (n = 3, Figure 3C). While the decrease in Western blotting was not significant, a linear trend with slope -0.5 was detected over the two-week course from day 7 to day 21 (p = 0.04). Adding to the finding of non-uniform staining in the airways, this suggests that undifferentiated cells have higher expression of SP-D.

MUC5AC, a mucin expressed in airway goblet cells, is used here as a marker for goblet cell differentiation. Immunohistochemistry of ALI cultures grown from non-asthmatic AEC demonstrated an increased expression of MUC5AC from day 7 through 21 (fraction of total epithelial area: 0.08 ± 0.02 7 days; 0.20 ± 0.06 14 days; 0.29 ± 0.05 21 days; n = 4, p = 0.007, Figure 3A and D). This indicated that differentiation into mucin-secreting goblet cells occurred in the ALI model throughout 21 days of culture.

CK-5, a marker for basal epithelial cells [3], is used to demonstrate the extent of differentiation in ALI cultures grown from non-asthmatic AEC. Immunohistochemistry on these cultures demonstrated significantly reduced expression of CK-5 (fraction of total epithelial area: 0.59 ± 0.04 7 days; 0.36 ± 0.07 14 days; 0.27 ± 0.05 21 days; n = 4, p = 0.002, Figure 3A and E). The decrease in CK-5 expression coordinate with decreased SP-D expression during differentiation suggests that the same CK-5 positive basal cells could potentially also be the main contributor of SP-D production in the airway epithelium.

### IL-13 effect on SP-D expression

**Figure 4:**
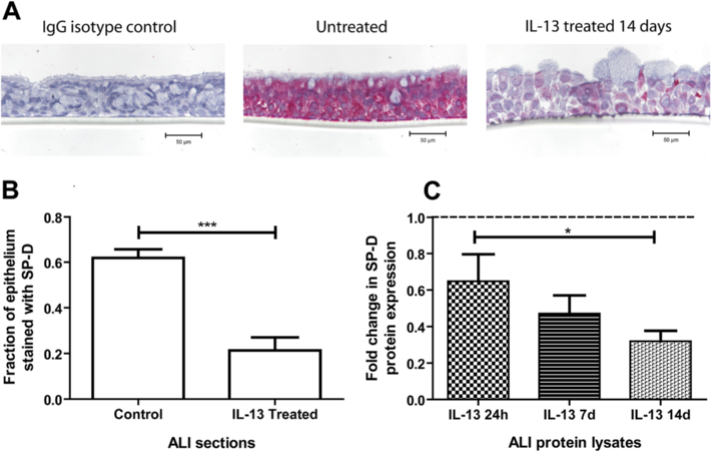
**Surfactant protein D (SP-D) expression in non-asthmatic ALI cultures treated with IL-13.** SP-D expression was examined in IL-13-treated (10 ng/ml, 14-day duration) ALI sections grown from matched non-asthmatic donors via immunohistochemistry, where pink was indicative of positivity **[A]**. Fraction of total epithelial area positive was quantified using colour segmentation in ImagePro Plus. Expression of SP-D in the airway epithelia decreased significantly in the cultures treated with IL-13 (n = 4, t-test p = 0.0005) **[B]**. SP-D protein expression in ALI cultures treated with IL-13 for 24 hours, 7 days, and 14 days were detected by Western blotting, quantified by densitometry, and normalized to untreated control to obtain fold changes. All three durations resulted in reductions of SP-D expression (n = 6, one-way ANOVA p = 0.02) **[C]**.

Two-week IL-13 treatment of ALI cultures grown from non-asthmatic AEC induced characteristic changes including altered epithelial thickness and cell type distribution (Figure 4A). With respect to SP-D expression, IL-13 induced a significant reduction in the fraction of epithelium that stained positive for SP-D (0.21 ± 0.06) compared to untreated (0.62 ± 0.04) as shown by immunohistochemistry (n = 4, p = 0.0005, Figure 4B). In a time-course experiment, IL-13 treatment of ALI cultures resulted in a fold reduction in SP-D protein expression relative to untreated cultures as demonstrated by Western blotting (0.65 ± 0.15 at 24 hours, 0.47 ± 0.10 at 7 days, 0.32 ± 0.06 at 14 days; n = 4-6, Figure 4C). A significant reduction was also found in SP-D protein expression at day 14 compared to day 7 (p < 0.05). Due to concurrent changes in cell growth, cell type, and epithelial structure, it is unclear whether this reduction is a direct effect of IL-13 or an indirect consequence of IL-13-induced changes in differentiation.

### IL-13 regulation of SP-D in monolayer culture

**Figure 5:**
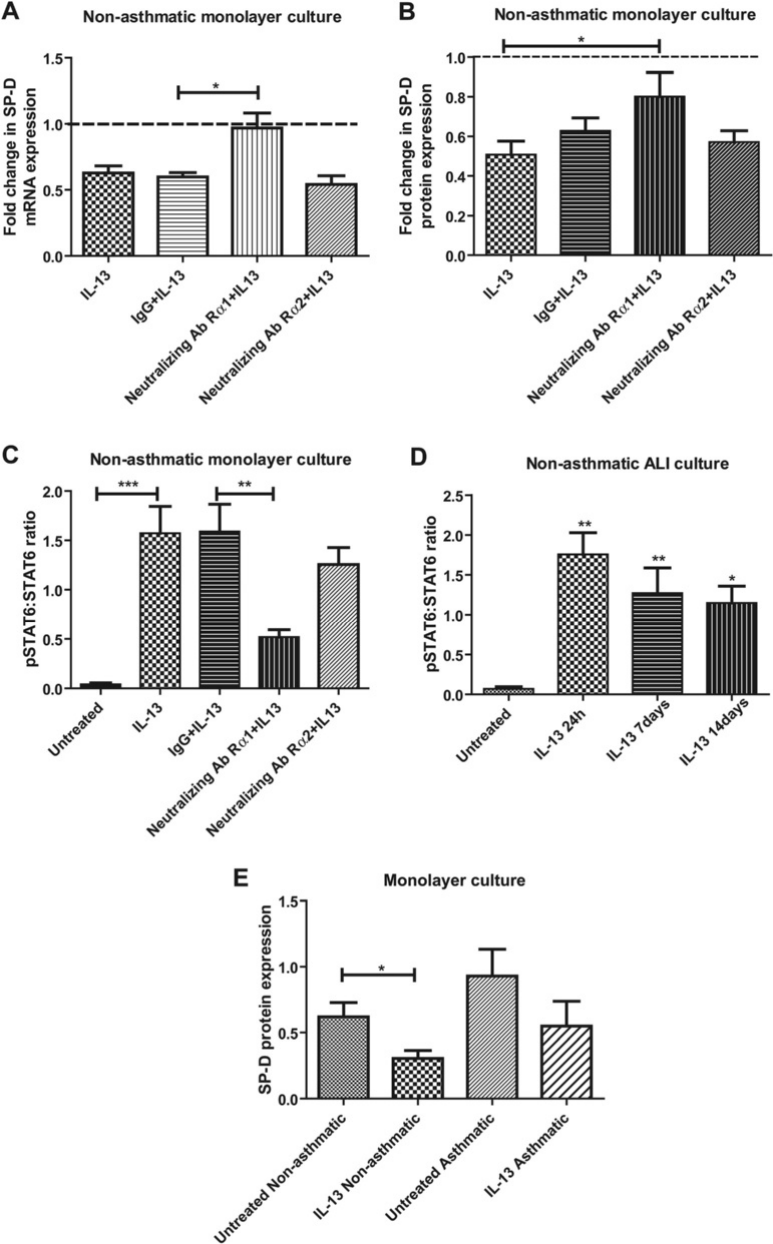
**SP-D expression in monolayer epithelial cultures treated by IL-13.** SP-D mRNA was examined in non-asthmatic monolayer cultures treated with IL-13 (10 ng/mL) alone and in cultures pre-treated with either goat IgG isotype control (20 μg/mL), IL-13Rα1 neutralizing antibody (20 μg/mL), or IL-13Rα2 neutralizing antibody (2 μg/mL) one hour prior to IL-13 exposure. To obtain fold changes, ΔΔ_CT_ values were obtained by normalizing SP-D mRNA to HPRT mRNA and normalizing treatment groups to untreated control. A significant difference was observed between the IgG pre-treated group and IL-13Rα1 neutralizing antibody pre-treated group (n = 3, one-way ANOVA p = 0.01) **[A]**. SP-D protein levels in non-asthmatic monolayer cultures under the same treatment conditions were detected via Western blotting. A significant difference was found between the IL-13 treated group and IL-13Rα1 neutralizing antibody pre-treated group. Quantification was performed by densitometry and SP-D protein expression was normalized to untreated control (n = 6, one-way ANOVA p = 0.04) **[B]**. Phosphorylated-STAT6 (pSTAT6) expression in non-asthmatic monolayer cultures from the same experiment as panel **[B]** was examined via Western blotting. IL-13 treatment significantly increased the phosphorylation of STAT6 compared to control while pre-incubation with IL-13Rα1 neutralizing antibody significantly reduced the phosphorylation of STAT6 compared to IgG (n = 5, one-way ANOVA p = 0.005) **[C]**. pSTAT6 to STAT6 protein ratio in non-asthmatic ALI cultures treated with IL-13 (10 ng/mL) over the course of two weeks show significant increases relative to untreated control (n = 3, one-way ANOVA p = 0.001) **[D]**. IL-13 treatment resulted in a significant decrease in SP-D protein expression in non-asthmatic monolayer culture (n = 5, t-test p = 0.03) whereas no significant change was detected in asthmatic monolayer culture (n = 4) **[E]**.

To study SP-D regulation in response to IL-13, monolayer cultures grown from non-asthmatic AEC were treated with IL-13 (10 ng/mL) for 24 hours. When normalized to untreated control, treatment with IL-13, either alone or after pre-incubation with IgG isotype control for 24 hours induced a reduction in SP-D mRNA (0.62 ± 0.05 and 0.60 ± 0.03 respectively, n = 3, Figure 5A). Cells pre-incubated with IL-13Rα1 neutralizing antibody prior to IL-13 treatment demonstrated a mitigation of the IL-13 effect on SP-D mRNA expression (0.99 ± 0.11). In contrast, cells pre-incubated with IL-13Rα2 neutralizing antibody demonstrated no significant difference in SP-D mRNA expression compared to cells pre-incubated with IgG isotype control (0.54 ± 0.07).

Total protein lysates collected from parallel experiments were analysed by Western blotting. Relative to untreated control, treatment with IL-13, either alone or after pre-incubation with IgG isotype control for 24 hours induced a reduction in SP-D protein (0.51 ± 0.08 and 0.65 ± 0.07 respectively, n = 6, Figure 5B). Pre-incubation with IL-13Rα1 neutralizing antibody prior to IL-13 demonstrated a similar mitigation of the IL-13 effect on SP-D protein expression (0.85 ± 0.13). Pre-incubation with IL-13Rα2 neutralizing antibody produced no significant difference in SP-D protein expression compared to either IL-13 treatment alone or IL-13 treatment with IgG pre-incubation (0.60 ± 0.06).

Activation of the transcription factor STAT6 was analyzed in matched protein lysates coordinate with SP-D expression to examine downstream activity of IL-13Rα1. The ratio of phosphorylated STAT6 to total STAT6 as a marker of activation was low in untreated control (0.04 ± 0.01), markedly increased in IL-13 treated cells (1.57 ± 0.27), in IgG pre-incubated cells (1.59 ± 0.28), and in IL-13Rα2 neutralizing antibody pre-incubated cells (1.25 ± 0.17) (n = 5, p = 0.005, Figure 5C). In contrast, pre-incubation with IL-13Rα1 neutralizing antibody resulted in a marked reduction in the ratio (0.52 ± 0.08).

STAT6 activation, studied in ALI cultures exposed to IL-13 for 24 hours, one week, and two weeks, was quantified by the phosphorylated STAT6 to total STAT6 ratio. This ratio was low in untreated ALI control (0.07 ± 0.03) and increased in IL-13 treatment groups (n = 3, Figure 5D). The highest ratio was observed at 24 hours (1.76 ± 0.27) which progressively decreased at 7 days (1.27 ± 0.32) and at 14 days (1.15 ± 0.21). This suggests that the cells continued to respond to IL-13 through IL-13Rα1 and the STAT6 pathway during the two weeks of exposure.

The effect of IL-13 on SP-D expression in asthmatic AEC was studied in monolayer cultures and compared with non-asthmatic AEC. Total protein lysates of untreated and IL-13-treated (10 ng/ml) asthmatic and non-asthmatic AEC were collected at 24 hours post-treatment and subsequently analysed by Western blotting. No significant difference in SP-D expression was detected in asthmatic AEC of the IL-13 treated group relative to untreated control cells (n = 4), whereas as significant reduction in SP-D expression was demonstrated in IL-13 treated (0.31 ± 0.06) non-asthmatic AEC relative to untreated control cells (0.62 ± 0.11) (n = 5, p = 0.03, Figure 5E).

## Discussion

In this study, we compared the expression of SP-D in airways of asthmatics to non-asthmatics and began to explore what may regulate this expression. The role of SP-D as a pattern recognition molecule in the innate immune system makes it relevant to asthma as multiple studies have demonstrated that asthma can occur subsequent to and be exacerbated by respiratory infections [7-9,22,23]. More recent studies have included the innate immune system, acknowledging that it may play important roles in the pathogenesis of asthma, affecting host susceptibility to infections and immune response to pathogens.

We demonstrated an elevated expression of SP-D in airways of asthmatics compared to that of non-asthmatics in lung tissue sections and ALI cultures, a model for epithelial differentiation. The increased expression of SP-D could arise from several possibilities: from structural differences, such as proliferation of a cellular subtype in the epithelium, to functional differences, such as over-production or lack of inhibition affecting gene expression of SP-D. Previously, Cheng *et al.* have shown elevated concentrations of SP-D in the bronchial alveolar lavage (BAL) of asthmatic patients compared to non-asthmatic controls; while a higher average concentration was found, the difference was not significant [24]. Koopmans *et al.* observed increased serum SP-D in allergic patients both at baseline and after allergen challenge [25]. Mouse models of chronic inflammatory conditions using *P. Carinii-*induced lung injury have found 7.4 fold and 71 fold increases of SP-D in BAL and serum respectively [26]. Murine models of acute allergic lung inflammation have demonstrated disparity in SP-D levels in BAL of C57BL/6 mice compared to BALB/c mice, suggesting a genetic component to the baseline production of SP-D in the lung [12].

SP-D gene deficient mice were found to exhibit hyper-eosinophilia and increased levels of IL-5 and IL-13 upon *Aspergillus fumigatus* allergen challenge, a response that was reversible by treating the mice with SP-D [27]. As SP-D participates in host defense and modulates inflammation, an increase in SP-D levels could potentially be beneficial if it plays a protective or even compensatory role in asthma and other chronic inflammatory conditions. While higher levels of SP-D in the airways of asthmatics seem counterintuitive in the context of increased susceptibility to viral infection in asthma, this suggest that underlying differences in the function of SP-D may exist in humans between asthmatics and non-asthmatics. In a study by Wang *et al.* purified SP-A and SP-D suppressed *Dermatophagonides pteronyssinus*-stimulated lymphocyte proliferation in naïve mice, but this suppressive effect was reduced in sensitized mice [28]. While the study was conducted in a murine model, this suggests that there may be dysregulated processes in the immunomodulatory roles of SP-D in sensitized patients such as those with asthma. With regards to SP-D function, Kishore *et al.* have proposed that SP-A and SP-D in naïve lungs can help mitigate potential damage from a low level of exogenous insults; however, when overwhelmed by high levels of insults, these collectins assume a pro-inflammatory role to complement innate and adaptive immunity [29].

Immunohistochemistry of ALI cultures demonstrated decreasing levels of SP-D expression as they differentiated over three weeks. Visual inspection led to the observation of SP-D in columnar cells and basal cells. Using MUC5AC as a marker for the presence of mucin-producing goblet cells, little to no SP-D staining was observed in goblet cells. Previously, Madsen *et al.* have localized SP-D in human lungs to alveolar type II cells, Clara cells, and on or within alveolar macrophages [30]. Kim *et al.* found surfactant proteins expressed in the ciliated cells of the nasal epithelium but not the goblet cells of human nasal mucosa [31]. Here we present novel data on characterization of SP-D within the airway epithelia of conducting bronchus. Our observations are consistent with the previous studies with regards to the lack of SP-D in goblet cells. Differences in SP-D expression within specific cell types could arise from the different regions of the respiratory tract studied.

In human airway sections, the more intense SP-D staining in basal cells relative to the remainder of the epithelium suggest that basal cells either produced more or retained more SP-D within their cytoplasm. This concurs with the observation of decreasing SP-D levels in the differentiating ALI cultures where the pluripotent basal cell, visualized with CK-5 as a marker, becomes significantly less abundant over 21 days of culture. In light of these observations, the increased SP-D expression in airways of asthmatics could be due to a greater proportion of undifferentiated cells. The increased number of goblet cells in asthma, which theoretically results in lower SP-D expression, may play a relatively minor role compared to the undifferentiated cells.

Treatment with IL-13 of non-asthmatic human airway epithelial cells, grown in both monolayer and ALI culture, resulted in decreased SP-D expression. Previous studies from our laboratory have demonstrated that IL-13 treatment of primary airway epithelial cells reduced spontaneous apoptosis in culture [32], thereby apoptosis-induced reduction of SP-D is unlikely. Studies to date have not come to consensus on how IL-13 modulates SP-D expression in the lungs. Haczku *et al.* found SP-D mRNA and protein levels in the lungs of mice increased in response to IL-4 and IL-13 treatment [33]. Ito and Mason found that IL-13 reduced mRNA and protein levels of SP-D in cultured human alveolar type II cells [34]. We have demonstrated that IL-4 and IL-6 (Additional file 1; Additional file 2: Figure S1), in addition to IL-13, all decrease SP-D expression by non-asthmatic human AEC. This discrepancy in SP-D response to IL-4 and IL-13 may be attributed to differences in species; for instance, a non-specific STAT-binding site is present in the SP-D promoter of rats and mice but not that of humans [35]. In contrast to the effect of IL-13 on non-asthmatic AEC, no significant difference was detected in SP-D expression between IL-13 treated and untreated asthmatic AEC. This suggests that asthmatic AEC have an altered response to IL-13 with regards to SP-D expression. This, in turn, may contribute to the increased SP-D observed in airway epithelium of asthmatics despite the elevated IL-13 levels in asthmatic airways as established by several groups [18,36-38].

Potential pathways for IL-13 regulation of SP-D were studied using monolayer cultures of human AEC. It has been shown that monolayer cultures have the same IL-13 receptors as ALI cultures [39]. Neutralizing antibodies for IL-13Rα1 and IL-13Rα2 were used to block the two receptors of IL-13. Data from both mRNA and protein demonstrate that IL-13 decreased the expression of SP-D and suggest that such a reduction was mediated through the IL-13Rα1 pathway as opposed to IL-13Rα2. We also demonstrated an increase in phosphorylated STAT6, a transcription factor downstream of IL-13Rα1, which is coordinate with the reduction in SP-D expression. Protein analysis of STAT6 phosphorylation affirmed that IL-13 treatment activated the STAT6 transcription factor and that this activation was to a large degree blocked by the pre-incubation with IL-13Rα1 neutralizing antibody.

Studies in monolayer cultures grown from non-asthmatic donors with exposure to IL-4 also demonstrated SP-D reduction and STAT6 phosphorylation. This supports the role of the heterodimeric receptor, consisting of IL4Rα and IL-13Rα1, in modulating SP-D levels (Additional file 1; Additional file 2: Figure S1). Treatment of monolayer cultures with IL-6 induced a decrease in SP-D as well (Additional file 2: Figure S1). While IL-6 signals through STAT3 [40], it is still possible that the pathways through which SP-D decreases in response to IL-13, IL-4, and IL-6 share common components.

Study of STAT6 activation on IL-13 treated ALI cultures grown from non-asthmatics demonstrated a drastic initial increase in the expression of phosphorylated STAT6 24 hours post-treatment followed by a gradual decrease over the span of two weeks. The sustained decrease in SP-D level over the same period suggests that the IL-13 effect on SP-D expression in non-asthmatic AEC is chronic and sustained.

Airway epithelium differentiation and IL-13 signalling are both implicated in the structural and functional changes associated with asthma. Hackett *et al.* have demonstrated that asthmatic patients have less differentiated airway epithelium [3]. This may be a consequence of persistent injury to the epithelium and result in an altered proportion of cell types as well as their protein expression profiles. IL-13 has been shown to mediate features of asthma independent of other cytokines in animal models [13,16,17]. While IL-13 is closely associated with allergic asthma and adaptive immunity, it is also valuable to know how it may affect molecules and pathways of innate immunity in asthma.

We have demonstrated that airway epithelia of asthmatics express more SP-D whereas non-asthmatic differentiated ALI cultures have decreases in SP-D expression in parallel with decreasing basal cells (via CK-5 marker) and increasing goblet cells (via MUC5AC marker). IL-13 treatment, which is known to induce goblet cell hyperplasia, reduced SP-D expression in non-asthmatic airway epithelial cultures via IL-13Rα1 but had no effect on SP-D expression in asthmatic airway epithelial cultures. This suggests that in airways of asthmatics, increased SP-D production is predominantly contributed by a less differentiated phenotype, while the increased number of goblet cells is negligible in affecting the expression of SP-D. Additionally, IL-13 regulation of SP-D expression in asthmatic airways was different from non-asthmatic airways, thus contributing to the observed disparity in the SP-D expression. Asthmatic patients being more susceptible to viral infections despite their increased SP-D expression leads one to question whether the SP-D molecules produced in these patients are less effective in their host defence roles.

Limitations of this study include the lack of functional data on SP-D. While SP-D expression levels were studied here, its function in binding pathogens and modulating immune response in the airways cannot be compared between people with and without asthma. Another limitation is related to the resolution of immunohistochemistry and the inability to clearly separate differentiated and undifferentiated cells of the airway epithelium. Visual inspection could only offer qualitative observations on the localization of SP-D. It is difficult to compare the contribution of SP-D expression in each cell type of the airway or in a model exhibiting the pseudo-stratified airway epithelium.

IL-13 signalling was studied in non-asthmatic AEC at the receptor level and any links, direct or indirect, between this and SP-D gene expression remains to be found. Future studies on SP-D can add depth to the molecular mechanisms in both asthmatic and non-asthmatic airways. These can provide insight into the role SP-D plays in asthma and sources from which dysregulation or dysfunction may arise with regards to innate immunity.

## Conclusion

Overall, this study demonstrated a difference in SP-D expression in airways of asthmatics relative to that of non-asthmatics. This can have implications on the increased susceptibility to infections and altered inflammatory response in asthmatic patients. There could be several factors contributing to the increased SP-D levels observed in the airway epithelium of asthmatics and further studies are required to elucidate the underlying mechanisms. Up-regulation of SP-D expression in asthma could be in response to the chronic inflammatory milieu of the airways. It may also be that SP-D molecules produced by asthmatics do not exhibit full functionality, hence cannot mediate inflammation or clear pathogens, and consequently production continues as the inflammation or infection persists. Another potential factor could be cell type and differentiation differences between the airway epithelium of asthmatics and non-asthmatics. Lastly, altered regulatory pathways through which SP-D expression is affected, such as IL-13, in the asthmatic airway epithelium may lead to airway remodelling differences.

## Additional files

Additional file 1:
**This includes the data obtained on monolayer cultures grown from non-asthmatic donors treated with IL-4, IL-6 or IL-13.**
Additional file 2: Figure S1. Graphical representation of SP-D levels in monolayer cultures grown from non-asthmatic donors treated with either IL-4 and IL-6 or IL-13.
