# Supplementary material for: Expression of surfactant protein D in airways of asthmatics and interleukin-13 modulation of surfactant protein D in human models of airway epithelium
Source: Respir Res. 2015 Feb 15;16(1):26. doi: 10.1186/s12931-015-0177-7 (PMC4352233; doi:10.1186/s12931-015-0177-7)
Supplement: Additional file 1: — This includes the data obtained on monolayer cultures grown from non-asthmatic donors treated with IL-4, IL-6 or IL-13. [file 12931_2015_177_MOESM1_ESM.doc]

**Introduction:**

Interleukins 4 and 6 (IL-4, IL-6), in addition to interleukin 13 (IL-13), are inflammatory cytokines which play roles in the pathogenesis of asthma . Previous studies have demonstrated elevated expression of SP-D induced by IL-4 in mice *in vivo* and *in vitro* . As IL-4 signaling pathway and effects on SP-D are closely linked with those of IL-13, studying its effects in the human airway would provide more insight as to how SP-D expression may be regulated by inflammatory cytokines such as IL-13. IL-6 have been demonstrated to be increased in non-symptomatic asthmatic patients and further increased in these patients during asthma attacks . It would also be value to study how these inflammatory cytokines, in turn, affect the expression of SP-D.

**Material and Methods:**

Recombinant human interleukin (IL) -4 (204-IL) waspurchased from R&D Systems (Minneapolis, MN). Recombinant human IL-6 (CHC1263 part 58.126.10) was purchased from Life Technologies (Burlington, ON).

Primary epithelial cells from asthmatic donors were grown to 80% confluence and stimulated for 24 hours IL-4 (30 ng/mL), IL-6 (100 ng/mL), and IL-13 (10 ng/mL). Total cell lysates were collected and used for Western blot analysis as described previously .

**Results:**

Stimulation of monolayer cell culture with IL-4, IL-13, and IL-6 reduced SP-D protein expression to 0.65 ± 0.04, 0.56 ± 0.06, and 0.78 ± 0.10 respectively relative to untreated control (n=3-5, Figure 1). As IL-4 and IL-13 could act through the same heterodimeric receptor, a decrease in SP-D as a result of stimulation by either suggest the receptor is a step in the pathway connecting the IL-4 and IL-13 with SP-D expression.

**Figures**

**Figure 1. SP-D expression in stimulated monolayer culture.** SP-D expression in non-asthmatic monolayer cultures treated with IL-4 (30 ng/mL), IL-13 (10 ng/mL), and IL-6 (100 ng/mL) for 24 hours was studied via Western blotting. Fold changes in protein expression were obtained by normalizing to untreated control and quantification was performed by densitometry (n=5).

**References**

**1. Chatila, T.A., *Interleukin-4 receptor signaling pathways in asthma pathogenesis.* Trends Mol Med, 2004. 10(10): p. 493-9.**

**2. Rincon, M. and C.G. Irvin, *Role of IL-6 in asthma and other inflammatory pulmonary diseases.* Int J Biol Sci, 2012. 8(9): p. 1281-90.**

**3. Haczku, A., et al., *IL-4 and IL-13 form a negative feedback circuit with surfactant protein-D in the allergic airway response*, in *J Immunol*. 2006: United States. p. 3557-65.**

**4. Cao, Y., et al., *IL-4 induces production of the lung collectin surfactant protein-D.* J Allergy Clin Immunol, 2004. 113(3): p. 439-44.**

**5. Yokoyama, A., et al., *Circulating interleukin-6 levels in patients with bronchial asthma.* Am J Respir Crit Care Med, 1995. 151(5): p. 1354-1358.**

**6. Patchell, B.J., et al., *Glycosylation and annexin II cell surface translocation mediate airway epithelial wound repair.* Am J Physiol Lung Cell Mol Physiol, 2007. 293(2): p. L354-63.**
