# Supplementary figures and images for: Expression of surfactant protein D in airways of asthmatics and interleukin-13 modulation of surfactant protein D in human models of airway epithelium
Source: Respir Res. 2015 Feb 15;16(1):26. doi: 10.1186/s12931-015-0177-7 (PMC4352233; doi:10.1186/s12931-015-0177-7)

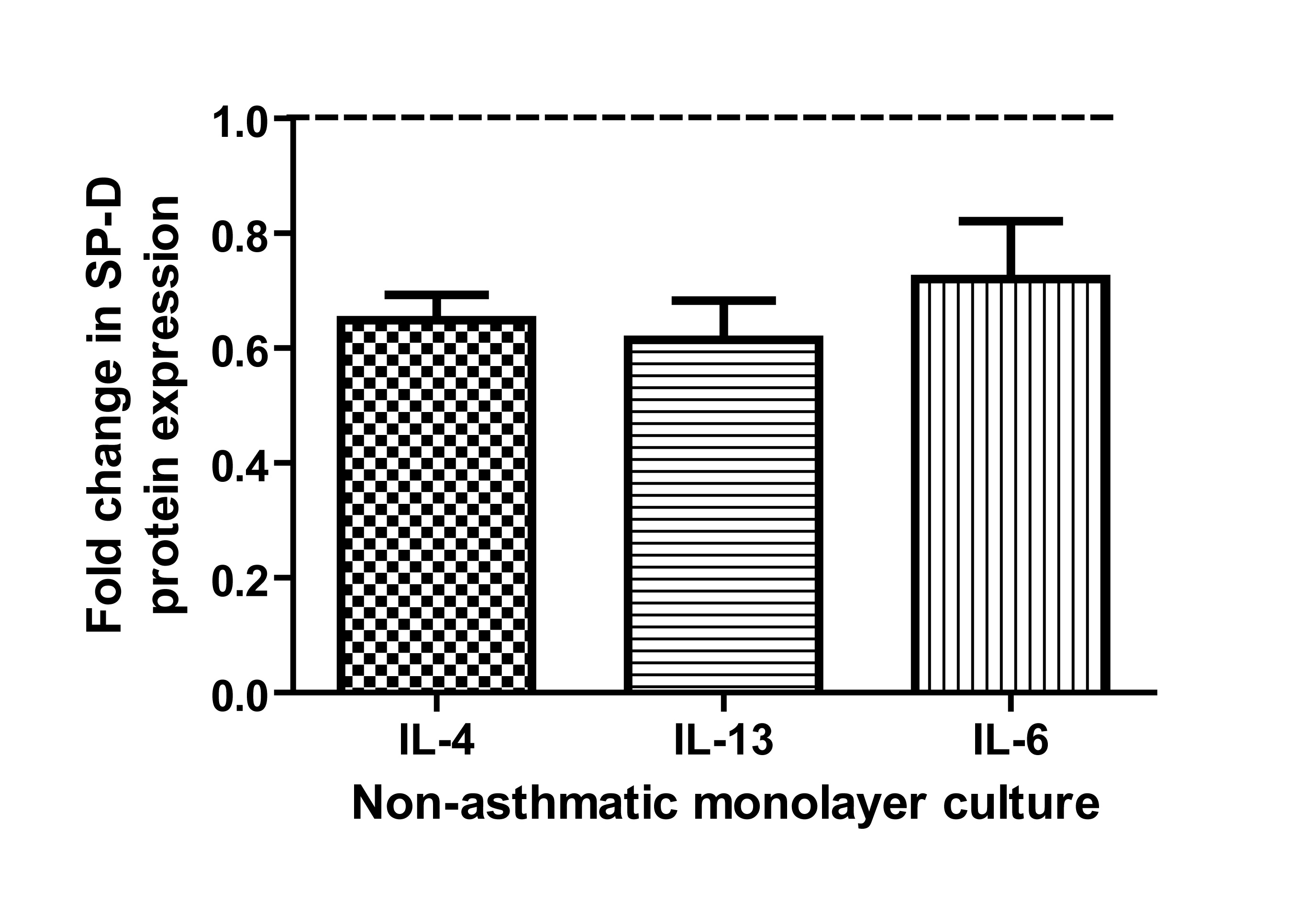

Supplement: Additional file 2: Figure S1. — Graphical representation of SP-D levels in monolayer cultures grown from non-asthmatic donors treated with either IL-4 and IL-6 or IL-13. [file 12931_2015_177_MOESM2_ESM.jpeg]
